# Integrated care for children and young people with special health and care needs: a systematic review

**DOI:** 10.1136/archdischild-2024-326905

**Published:** 2024-07-30

**Authors:** Swapnil Ghotane, Raeena Hirve, Julia Forman, Daniel Tan, Zak Achercouk, Ingrid Wolfe

**Affiliations:** 1 Department of Women & Children's Health, King's College London, London, UK; 2 University of Cambridge, Cambridge, UK

**Keywords:** Child Health, Child Health Services

## Abstract

**Context:**

There is a dearth of high-quality evidence on integrated, coordinated and cost-effective care for children with special health and care needs (CSHCN).

**Objective:**

To assess the effectiveness of integrated/coordinated care models for CSHCN.

**Data sources:**

Embase, Ovid Medline(R), HMIC Health Management Information Consortium, Maternity & Infant Care Database (MIDIRS), PsycARTICLES, PsycINFO, Social Policy and Practice, Cochrane Central Register of Controlled Trials (CENTRAL), Global Health and PubMed.

**Study selection:**

Inclusion criteria comprised (1) randomised trials, including cluster randomised trials; (2) an integrated/coordinated care intervention; (3) for children and young people under 25 with special healthcare needs including medical complexity; (4) assessing child-centred outcomes, health-related quality of life among parents and carers, and health or social care use, processes of care and satisfaction with care.

**Data extraction:**

Data were extracted and assessed by two researchers, and descriptive data were synthesised according to outcome and intervention.

**Results:**

14 randomised controlled studies were included. Seven out of the 14 studies had a dedicated key worker/care coordinator as a vital part of the integrated/coordinated care intervention; however, the certainty of evidence for all outcomes was either ‘low’ or ‘very low’.

**Limitations:**

Included studies were mostly from high-income countries. Variable study outcomes and quality of evidence precluded meta-analysis.

**Conclusions:**

Limited evidence favours integrated care for CSHCN using a dedicated key worker/care coordinator; however, heterogeneity in study outcomes and definitions of CSHCN limit the strength and utility of evidence obtained. Recommendations are made for improving integrated care practice, research and evaluation which are important for evidence-based health services for CSHCN.

**PROSPERO registration number:**

CRD42020209320.

**WHAT IS ALREADY KNOWN ON THIS TOPIC**

- Children with special health and care needs (CSHCN) have a broad scope and variety of complex needs; however, most integrated care models are based on specific medical conditions or types of medical complexity.

**WHAT THIS STUDY ADDS**

- Considering both medical and non-medical needs, the findings provide limited evidence of benefits, for CSHCN, of integrated care models with a key worker or care coordinator.
- There is a lack of high-quality evidence and standardised outcomes for research about CSHCN.

**HOW THIS STUDY MIGHT AFFECT RESEARCH, PRACTICE OR POLICY**

- The potential for integrated care, specifically care coordination, for CSHCN should be investigated with high-quality research, robust study designs, consistent outcome measures and across diverse settings.

## Introduction

 Children with special health and care needs (CSHCN), including chronic conditions, medical complexity and disability, have high levels of needs and rely on a range of services across sectors.^1^ In the USA, the prevalence of children under 18 years with special healthcare needs increased from 12.8% (about 9.4 million) in 2001 to around 19% (about 14 million) in 2022.^2 3^ In England, approximately 86 625 children between 0 and 19 years were reported to have a life-limiting condition in 2017–2018.^4^ The impact of chronic conditions on children is considerable including mortality^5^ and lower health-related quality of life.^6^

### Integrated care for CSHCN

Globally, there is a growing health policy interest in developing integrated care models especially in high-income countries as seen in Europe,^7^ the UK^8^ and the USA.^9^ Definitions of integrated care vary^10^; this review uses the WHO definition, ‘integrated care is concentrated on the needs of individuals, their families and communities in order to improve patient experience and achieve greater efficiency and value from health delivery systems’.^11^

**Figure 1:**
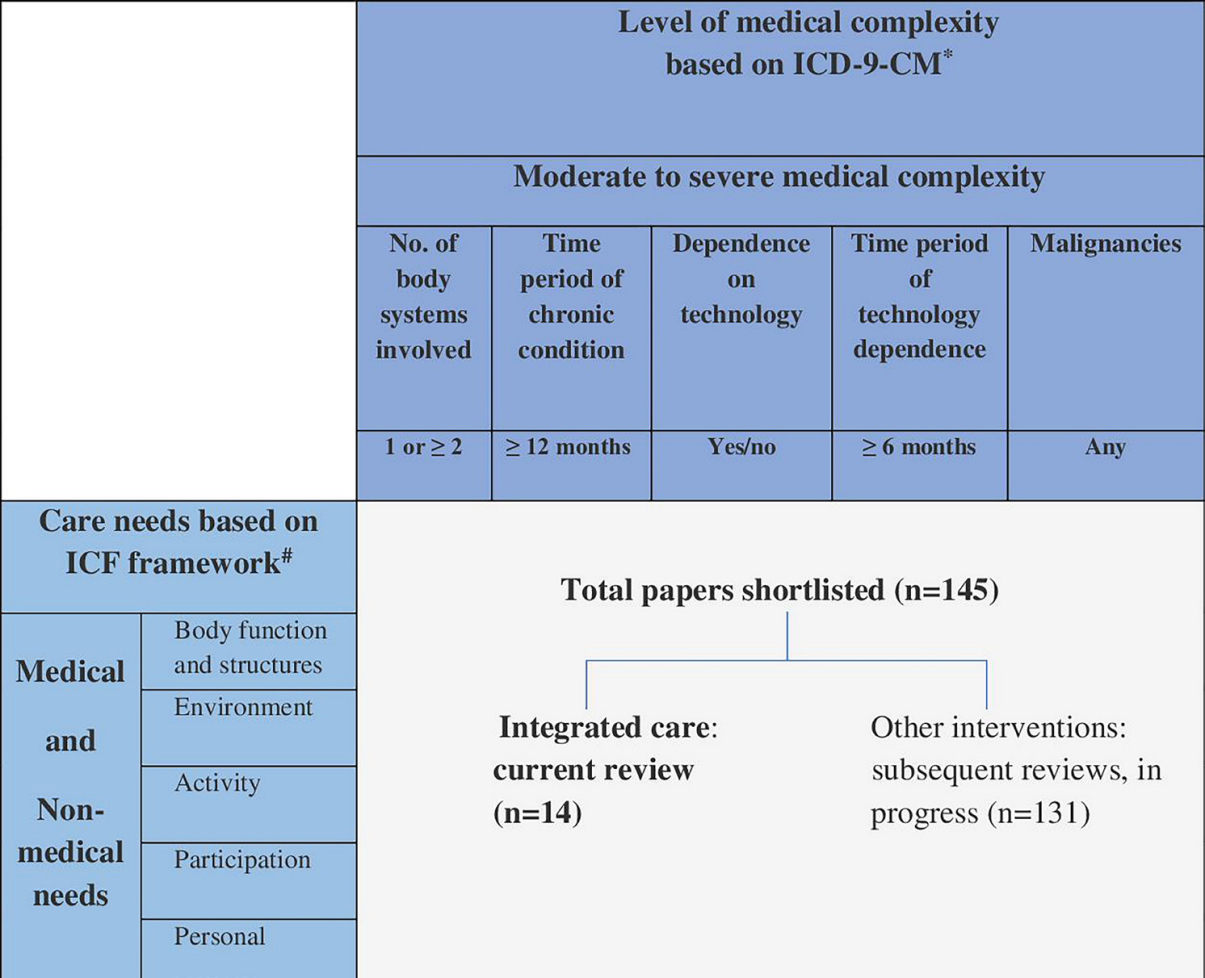
Framework of care needs and medical complexity for mapping evidence on integrated care. ^#^World Health Organisation^14^+Glader *et al.*^15^ *Simon *et al*.^16^

Previous studies have explored evidence on integrated care; however, a majority of those are for children with specific medical conditions or medical complexity^12^ or about narrowly defined care models.^13^ Given the heterogeneity and broad scope of complex medical and non-medical needs among CSHCN, it is perhaps not surprising that there is a lack of evidence about integrated care. However, given the policy prominence, we sought to provide a comprehensive overview of evidence. We began by considering two established sets of criteria: care needs as defined by the WHO ‘International Classification of Functioning, Disability and Health’ (ICF) framework^14 15^ and level of medical complexity defined by Simon *et al*.^16^ We used these criteria (figure 1) to describe evidence gaps for integrated care and related interventions and plan a programme of work. This systematic review addresses an important gap by assessing the evidence about integrated care to meet medical and non-medical needs for CSHCN.

## Methods

The protocol was developed according to the Preferred Reporting Items for Systematic Reviews and Meta-Analyses-Protocol (PRISMA-P) guidance^17^ and was registered in PROSPERO: CRD42020209320.

### Search strategy and data sources

The search strategy was developed in consultation with paediatric clinicians and experts in systematic review methodology (online supplemental appendix 1). We searched 10 databases, restricted to English language publications, from inception to 30 May 2023 (online supplemental appendix 1).

### Inclusion and exclusion criteria

We included studies eligible by as follows: *Population*: studies involving children and/or young people below 25 years with a chronic medical condition lasting for at least a year, with medical and non-medical needs (at least one) including activity, environmental, personal or participation factors. *Intervention*: integrated care models, care coordination or care programmes. *Outcomes*: primary outcomes were (1) child-centred outcomes relating to physical, functional or mental health status, or relating to behaviour or school attendance, and (2) effectiveness of integrated care or care coordination. Secondary outcomes included (1) health-related quality of life and well-being of parent/guardian/carer and (2) health and social care use, processes of care/satisfaction of care. *Study type*: randomised controlled trials (RCTs) including cluster randomised trials where the comparison (control) was standard or routine care. We excluded all other study designs.

### Data management

All titles, abstracts and full-text articles were reviewed (by SG and RH) and data were extracted by four review authors (SG, RH, DT and ZA). Discussions with a third author (JF) were used to resolve any disagreements. The following study characteristics were extracted: study design, study setting, intervention components, participant demographics, outcomes, key findings and conclusions.

Two authors (SG and RH) independently assessed the risk of bias for each study using the Cochrane risk-of-bias assessment tool^18^ and evidence certainty using the Grading of Recommendations, Assessment, Development and Evaluation (GRADE) criteria.^19^ They (SG and RH) also manually examined the references in the short-listed articles.

### Analysis

A narrative synthesis of evidence about integrated care to meet medical and non-medical needs of CSHCN was conducted, according to outcome and intervention. Heterogeneity of conditions and needs meant that meta-analysis was not feasible.

## Results

### Search results

**Figure 2:**
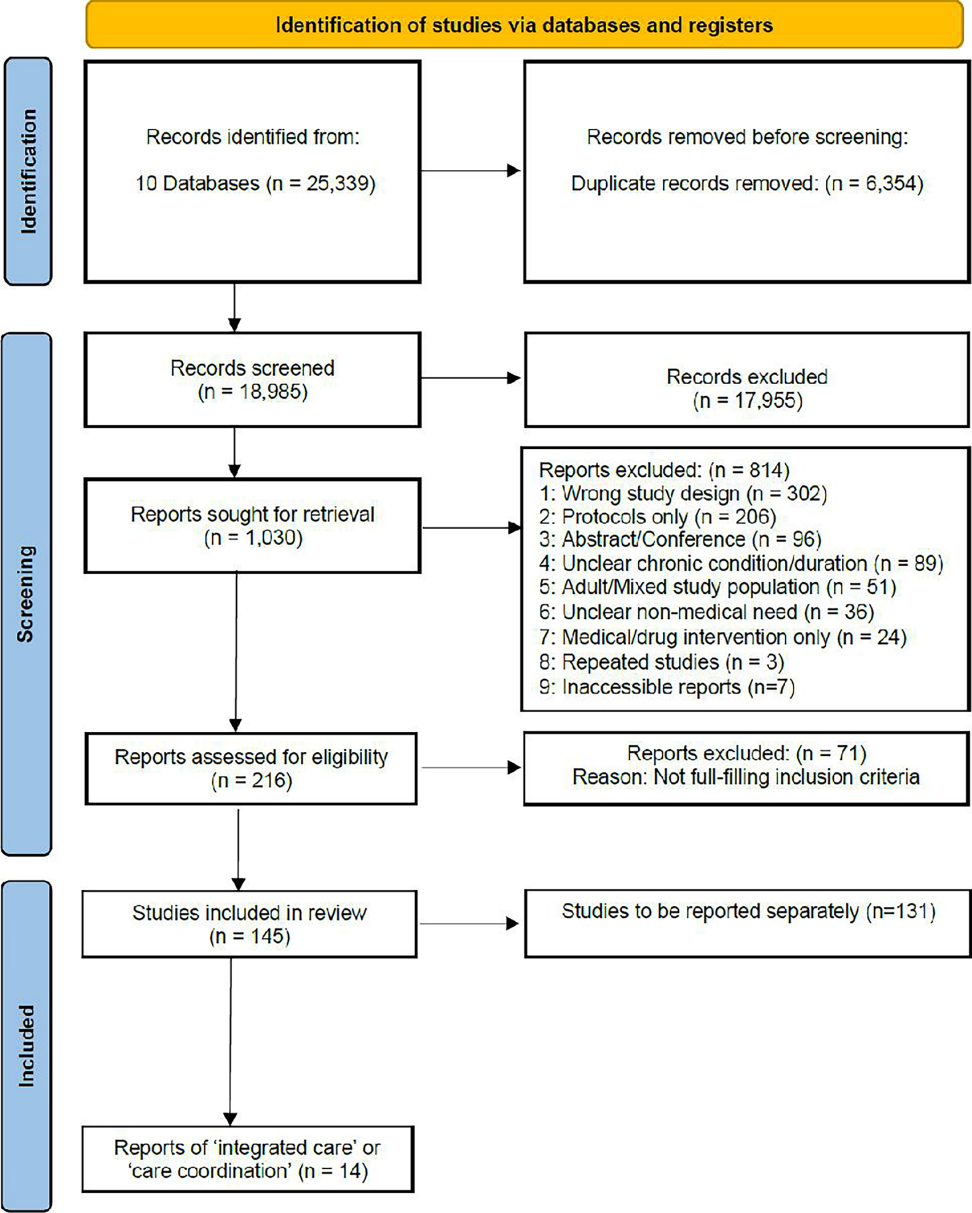
PRISMA (Preferred Reporting Items for Systematic Reviews and Meta-Analyses-Protocol) flow chart process of literature search. Adapted from Page MJ, *et al*.^42^http://www.prisma-statement.org/

The search generated 18 985 articles which were eligible for review of title and abstract (figure 2). About 1030 articles were eligible for full-text review and, subsequently, of which 814 studies did not meet the inclusion criteria. Further, 216 articles were selected for data extraction, of which 71 studies were then excluded, as on closer scrutiny did not to meet the inclusion criteria. Hence, a total of 145 studies were finally included which were then categorised by type of intervention. Applying our framework (figure 1), we identified 14 studies of integrated care interventions and the remaining 131 studies which assess other interventions will be published separately, producing a suite of reviews of evidence-based interventions for CSHCN.

### Study characteristics

There were 10 studies in which the chronic condition involved multiple disorders ranging from motor, mental, behavioural or emotional to neurodevelopmental conditions,^20^,^29^ and one study each about children with type 1 diabetes (T1D) and type 2 diabetes (T2D),^30^ medically fragile infants,^31^ behaviour problems with comorbid Attention Deficit Hyperactivity Disorder (ADHD)^32^ and traumatic brain injury (TBI).^33^ The number of participants in intervention and comparison groups were usually balanced in number and by gender, and the ages of study participants varied between 0 and 25 years. Only 4 out of 14 studies, namely, Cady *et al*,^21^ Cohen *et al*^25^*,* Caskey *et al*^27^ and Frakking *et al,*^28^ were found to have a ‘low’ overall ‘risk of bias. The GRADE analysis of evidence certainty was either ‘low’ or ‘very low’ for all outcomes, as shown in table 1.

**Table 1:** Characteristics of studies, outcomes and interventions

Study(Author) (Year)^ref^ (Country)	Chronic and complex condition(s) included in study	Totalparticipants (N)andrisk of bias score	Outcomes( 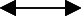 = no difference; 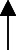 = improvement; = 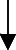 decline)(p value)^Scale/Instrument for outcome^(GRADE score)				Description of integrated care/care coordination	WHO integrated care model category
			Child health and functioning	Parent/carer perception on child health and family well-being	Healthcare use and spend	Care coordination		
Beyene *et al* 2013^20^USA	Multiple disorders	445 and moderate		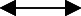 (p>0.05)^i,j,m,q^*Very low*			Children’s Treatment Network: an integrative health team together with family members develop a tailored single plan of care for the child, through *a* **service coordinator**	Managed clinical network
Braga *et al* 2005^33^Brazil	TBI	72 and high	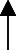 (p=0.018)^a^ Low(p=0.05)^c^*Very low*				Indirect, family-supported treatment: to provide families the skills to deliver home-based care through two case managers with appropriate specialisations to train families and acting as **service navigators**	Case management
Bruns *et al* 2015^26^USA	Multiple disorders	93 and high	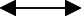 (p>0.05)^e, f^*Very low*				Wrap-around service of individualised, team-based holistic care planning through wraparound facilitators acting as **service navigators**	Care planning
Cady *et al* 2015^21^USA	Multiple disorders	163 and low				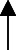 (p<0.05)^y^*Very low*	A single clinic-based **advanced practice registered nurse care coordinator**	Care planning
Carcone *et al* 2015^30^ USA	Type 1 diabetes and type 2 diabetes	146 and high				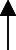 (p<0.05)^z^*Very low*	Multisystemic therapy: an intensive, home and community-based family treatment (psychotherapy approach), delivered by a **psychotherapist** functioning as a care coordinator	Care planning
Caskey *et al* 2019^27^ USA	Multiple disorders	6259 and Low			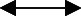 (p>0.05)^w, x^*Very low*		CHECK (Coordinated HEalthcare for Complex Kids): quality improvement project of comprehensive community-based care for children and young adults with care coordination delivered by **community health workers**	Disease-specific integrated care model
Cohen *et al* 2023^25^Canada	Multiple disorders	139 and low	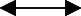 (p>0.05)^h^*Very low*	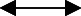 (p>0.05)^¶,n, **s, t,** u, v^*Very low*	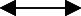 (p>0.05)^w, **x**^*Very low*	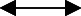 (p>0.05)^$, ¥^*Very low*	Complex Care for Kids Ontario (CCKO): Assignment of a **nurse practitioner–paediatrician dyad** partnering with families in a structured complex care clinic to provide intensive care coordination and comprehensive plans of care	Managed clinical network
Coller *et al* 2018^22^USA	Multiple disorders	147 and moderate			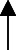 (p=0.04)^w^(p=0.02)^x^*Very low*		Plans for Action and Care Transitions, delivered by multidisciplinary team, created by **a medical home physician or nurse practitioner**	Care planning
Farmer *et al* 2011^23^USA	Multiple disorders	70 and moderate	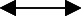 (p>0.05)^g1^*Very low*	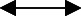 (p>0.05)^k, o, r^*Very low*	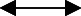 (p>0.05)^€^*Very low*		**Family support specialist**: liaising with primary care, specialty healthcare providers and community service agencies to improve access to and coordination of comprehensive care	Care planning
Frakking *et al* 2021^28^ Australia	Multiple disorders	81 and low		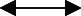 (p>0.05)^i,l, p^*Very low*			Care coordination delivered by an **allied health liaison officer**	Care planning
Gillette *et al* 1991^31^ USA	Medically fragile infants	38 and high	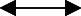 (p>0.05)^b, d^*Very low*			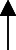 (p<0.001)^$^*Very low*	CATCH: A Collaborative Approach to the Transition from the Hospital to the Community and Home through a collaboration of **family and the community professional**	Case management
Jessop *et al* 1994^24^ USA	Multiple disorders	174 and moderate				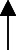 (p<0.05)^£^*Very low*	Paediatric home care: a comprehensive outreach programme in which a **multidisciplinary team** of paediatricians, paediatric nurse practitioners, and a social worker deliver comprehensive services, including case management	Patient-centred medical home
Kolko *et al* 2020^32^USA	Behaviour problems with comorbid ADHD	206 and moderate		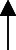 (T1-p=0.03)^i^(T2-p=0.02)*Very low*			Doctor Office Collaborative Care (DOCC)—Children and parents received on-site services in coordination with the primary care providers through **care managers**	Case management
Simon *et al* 2017^29^USA	Multiple disorders	331 and moderate	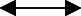 (p>0.05)^g2^*Very low*		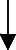 (p>0.05)^w, x^*Very low*	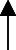 (T1-p=0.05) (T2-p=0.004)^✞^*Very low*	Seattle Children’s Hospital (SCH) developed a comprehensive case management service, a multidisciplinary, hospital-based service focused on improving care coordination for CMC	Case management

Note: See online supplemental table 1. online supplemental table 1 for full details.

ADHDAttention Deficit Hyperactivity DisorderCMCchildren with medical complexityGRADEGrading of Recommendations, Assessment, Development and EvaluationTBItraumatic brain injury

### Interventions

Out of the 14 studies, 7 are of interventions in which integrated care revolves around a designated person, including a care coordinator,^21^ family support specialist,^23^ a nurse practitioner–paediatrician dyad,^25^ community health workers,^27^ allied health liaison officer,^28^ a care manager^32^ and a psychotherapist,^30^ while in the remaining 7 studies, integrated care is delivered via a multidisciplinary team (table 1).

Using the WHO’s integrated care models framework classifications,^11^ six studies^21^,^2326 28 30^ included care planning models, with shared care plans and care coordinators. Four studies^2931^,^33^ were of interventions categorised as case management in which coordination of care is delivered by the case manager. The study by Jessop and Stein^24^ is a patient-centred medical home model, described as ‘a physician-directed group practice that can provide care which is accessible, continuous, comprehensive and coordinated and delivered in the context of family and community’.^11^ The study by Caskey *et al*^27^ is classified as a disease-specific integrated care model, as it targeted children and youth under the age of 25 with either asthma, diabetes (type 1 or 2), prematurity, seizure disorder or sickle cell disease. Finally, the study by Ye *et al*^20^ and Cohen *et al*^25^ was considered as a ‘managed clinical network model’ in which local health professionals and organisations work together, without being constrained by professional boundaries, to deliver quality care.^11^

### Outcomes

The outcomes measured varied between the 14 studies, with four overarching categories: *child health and functioning*,^23 25 26 29 31 33^ *parent/carer perception on child health and family well-being*,^20 22 23 25 28 32^ *healthcare use and spend*^22 25 27 29^ and *care coordination*^2123^,^25 29^ (table 1).

Within the *child health and functioning* category, improvement was seen in all outcomes^23 25 26 29 31^; however, significant improvement was reported only by Braga *et al*^33^ for both motor and cognitive outcomes, respectively. Similarly, for outcomes under the *parent/carer perception on child health and family well-being* category, improvements were reported for all outcomes^20 23 25 28^ and statistical significance was achieved in only one study^32^ for child’s psychosocial quality of life (QoL).

Within the *healthcare use and spend* category, two studies showed reduction in hospitalisation rate,^22 27^ although it was significant only in Coller *et al.* (adjusted incident rate ratio: 0.61; 95% (CI) 0.38–0.97; p=0.04)).^22^ Whereas for hospital admissions, one study reported reduction^25^ and the other reported slight increase^29^ (for both studies p>0.05).

In terms of total charges associated with patients, one study showed significant reduction (p=0.02)^22^ in the intervention group, one study reported reduction in both the intervention and control groups (p=0.99),^27^ and one study reported lower costs for the control group (p=0.90).^25^ In contrast, higher ‘health plan and clinic’ costs were reported by Simon *et al*^29^ for the intervention group at both 12 (p=0.09) and 18 months follow-up (p=0.01).

For *care coordination*, the studies by Carcone *et al*,^30^ Gillette *et al*,^31^ and Jessop and Stein^24^ reported a significant improvement (p<0.05), whereas the study by Farmer *et al*^23^ reported a strong trend (p=0.058) towards improved ratings of ‘satisfaction with care coordination’. Two studies^21 29^ reported significantly (p<0.05) more ‘help’ received by the intervention group for coordinating with multiple providers. Furthermore, the study by Cohen *et al*^25^ reported no significant differences in perceived care coordination among healthcare professionals or between clinicians and families.

## Discussion

This systematic review presents a narrative synthesis of evidence from 14 RCTs of integrated care interventions for CSHCN. Nearly all studies (n=12), regardless of the WHO’s type of integrated care model they represent,^11^ reported benefits of integrated care, with 8 studies reporting significant positive impacts on outcomes.^2122 24 29^,^33^ Only Bruns *et al*^26^ did not report any improvement for CSHCN using wrap-around care facilitators, and Simon *et al*^29^ reported higher hospitalisation admissions and costs in the group receiving the intervention.

Previous research has emphasised the importance of care coordination as well as continuity of care through a designated or dedicated personnel especially for CSHCN.^34^ In this research, seven studies^21 23 25 27 28 30 32^ exclusively described the role of a designated person in charge of care coordination, of which three reported significant improvement in *care coordination*,^21 23 30^ *child health and functioning*^32^ and a positive trend in *healthcare use and spend*^27^ (although not statistically significant). However, caution in interpreting the findings is needed given the inconsistencies across these studies. This is also echoed through the findings of a systematic review^35^ exploring evidence exclusively on the role of a care coordinator for CSHCN which highlighted the need for further evidence evaluating the role of care coordinators. Nevertheless, the findings from this review support recent NICE (National Institute for Health and Care Excellence) guidelines for children and young people (CYP) with disability up to 25 years of age with severe complex needs which recommends local authorities adopt a key working approach, preferably through a ‘dedicated key worker’ providing CYP and their families with a single point of contact to help ensure holistic provision and coordination of services.^36^

This review also highlights the lack of consistent (or core) outcome measures which presents a complex challenge for this topic. For instance, seven studies reported on care coordination^2123^,^25 30 31^ as either a primary or secondary outcome; however, they used different measures contributing to the ‘very low’ certainty of evidence. In addition, there was a lack of consistent tools used among the four categories of outcomes as all outcomes were measured using a unique instrument except for parent’s perception on child’s QoL reported by four studies,^20 25 28 32^ of which three used the same instrument (Pediatric Quality of Life—PedsQL)^20 28 32^ and one used KIDSCREEN-52.^25^

Although the findings on care integrated care are encouraging, it is important to consider the following challenges. First, that integrating care can be intensive and costly, sometimes with no measurable improvement in child functional status or hospital-based utilisation.^29 37 38^ However, current evidence also fails to distinguish between potential explanations such as whether integrated care improves access universally leading to increased service use and costs, and/or whether integrated care uncovers unmet needs^39^ and thereby appropriately leads to higher costs. This matters potentially a great deal since the former could lead to inadvertent widening of inequalities (as new interventions may often do), while the latter could be leading to genuine public good, despite increased costs.

### Strengths and limitations

A novel strength of this work is the focus on both medical and non-medical interventions which should be helpful for policymakers. Including RCTs only is both a strength (rigorous study design) and a weakness (it misses other useful study designs). While we acknowledge that there are many studies evaluating integrated care for CSHCN using other study designs, the quality is variable and sometimes poor.

A further potential weakness is in population heterogeneity and definitions. We tried to address these problems by focusing and simplifying the evidence as described in figure 1. A meta-analysis, however, was not possible, and hence, a narrative synthesis was presented. Out of 14 studies, 13 were based on high-income settings (USA: n=11^20^,^2427 29^; Canada: n=1^25^; Australia: n=1^28^) with a single study from a middle-income setting (Brazil)^33^; also, the search was limited to English language publications only; thus, the findings may have limited generalisability across countries and health systems. Furthermore, inadequately described interventions meant that some assumptions had to be made. For example, among the 14 short-listed studies, only 7 studies^2123^,^25 29^ reported ‘care coordination’ as either a primary or secondary outcome, whereas the rest of the studies implied that improvement in outcomes related to child health, parent’s perception or satisfaction, or healthcare use, could result from better care coordination.

## Conclusion

The study findings suggest limited evidence of the advantages of incorporating a key worker or care coordinator for CSHCN in integrated care models. This could be attributed to the stark lack of high-quality evidence and absence of a minimum set of standardised outcomes which could be generalised across populations of CSHCN. Our findings underscore the importance of further research with high-quality study designs, clearly defined populations and interventions, and consistent outcome measures, and including studies from low- and middle-income settings.

### Further research

We conclude with recommendations to improve integrated care practice, research and evaluation for CSHCN.

- An agreed set of core outcome measures of effectiveness of integrated care and care coordination for CSHCN is needed as this gap reduces utility of the existing evidence.^40^
- Comprehensive pragmatic trials are feasible to be conducted at scale, as we have recently demonstrated^41^ to explore the impact of complex system level interventions. This would be especially useful for CSHCN.
- Nested high-quality qualitative research is essential for detailed and nuanced study of the impact of interventions.
- Detailed and well-conducted process evaluations are critical to understanding the impact or lack of impact of complex interventions.
- Analysis of trends and social patterns of intervention uptake, and of residual population needs, is required to explore the complex problems of whether integrated care appropriately increases costs by meeting unmet needs.

## supplementary material

10.1136/archdischild-2024-326905 online supplemental file 1

10.1136/archdischild-2024-326905 online supplemental table 1

## Data Availability

Data sharing not applicable as no datasets generated and/or analysed for this study.
