# Supplementary material for: Integrated care for children and young people with special health and care needs: a systematic review
Source: Arch Dis Child. 2024 Jul 30;109(11):e326905. doi: 10.1136/archdischild-2024-326905 (PMC11503122; doi:10.1136/archdischild-2024-326905)
Supplement: online supplemental file 1 [file archdischild-109-11-s001.pdf]

Appendix 1. Search strategy

HMIC Health Management Information Consortium, Maternity & Infant Care Database (MIDIRS), PsycARTICLES, PsycINFO, Social Policy and Practice, Cochrane Central Register of Controlled Trials (CENTRAL), Global Health and PubMed

AND	**A. Target group**	adolescen* OR "Adolescents and Young Adults" OR "AYA" OR bab* OR child* OR infan* OR minor? OR neonat* OR p?ediatric OR puberty OR pubescent OR teen OR young* OR youth OR "young people"
	**B. Complex/ special care needs/ Multi/ comorbidity/disability**	"chronic care" OR "chronic condition?" OR "chronic disab*" OR "chronic disease?" OR "chronic health issue?" OR "chronic illness" OR "chronic impairment" OR "chronic mental issue?" OR "chronic mental condition?" OR "chronic mental disease?" OR "chronic psych*" OR "child disab*" OR "child medically fragile" OR "children with medical complex*" OR "children with special health care need?" OR "children with special health care need?" OR "chronic health conditions in childhood" OR "co-morb*" OR "complex care" OR "complex chronic condition?" OR "complex health need?" OR "development* disab*" OR disab* OR "life-limiting condition?" OR "life-long care" OR "life-long condition?" OR "life-long disease?" OR "life-long illness" OR "life-long impairment" OR "life-threatening condition?" OR "life-threatening disease?" OR "long term care" OR "long term condition?" OR "long term disease?" OR "long term health" OR "long term illness" OR "long term impairment" OR "long term psych*" OR "long term mental issue?" OR "long term mental condition?" OR "long term mental disease?" OR "long term psych*" OR "medical technology dependent" OR "medical complex*" OR "multi-morb*" OR "profound intellectual and multiple disabilities" OR "Severe motor and intellectual

		disabilities" OR "severe motor and intellectual disabilities medical care dependent group" OR "special health* need*" OR "special health care need?" OR "special need*" OR "Special Needs Kids" OR "SpeNK" OR "CSHCN" OR "CMC" OR "CCMC" OR "CYSHCN" OR "PIMD" OR "SMID" OR "SMID-MCDG"
	**C. Integrated Care Model**	"care continuity" OR "care coordination" OR "care framework" OR "care pathway" OR "care planning" OR "care transition" OR "case management" OR "care model" OR "child health services" OR "chronic care model" OR "collaborative care" OR "continuity of care" OR "cooperative care" OR "coordinated care" OR "coordinated care model" OR "disease management" OR "horizontal integration" OR "integrate* care" OR "interdisciplinary" OR "interprofessional" OR "intersectoral care" OR "intrasectoral care" OR "linked care" OR "longitudinal integration" OR "managed care" OR "medical home" OR "multi sector" OR "multiagency" OR "multidisciplinary" OR "multiprofessional" OR "patient centered" OR "self-care" OR "service network" OR "shared care" OR "transitional care" OR "vertical integration" OR "virtual integration" OR "whole system thinking"
	**D. Randomized controlled trials**	**The Cochrane HSSS strategy (filter)**<br>1. randomized controlled trial.pt.<br>2. controlled clinical trial.pt.<br>3. randomized.ab.<br>4. placebo.ab.<br>5. clinical trials as topic.sh.<br>6. randomly.ab.

		7. trial.ti.<br>8. 1 or 2 or 3 or 4 or 5 or 6 or 7<br>9. exp animals/ not humans.sh.<br>10. 8 NOT 9

Database: Embase <1947 to 2023 May 30>:

AND	**A. Target group**	adolescen* OR "Adolescents and Young Adults" OR "AYA" OR bab* OR child* OR infan* OR minor? OR neonat* OR p?ediatric OR puberty OR pubescent OR teen OR young* OR youth OR "young people"
	**B. Complex/ special care needs/ Multi/ comorbidity/disability**	"chronic care" OR "chronic condition?" OR "chronic disab*" OR "chronic disease?" OR "chronic health issue?" OR "chronic illness" OR "chronic impairment" OR "chronic mental issue?" OR "chronic mental condition?" OR "chronic mental disease?" OR "chronic psych*" OR "child disab*" OR "child medically fragile" OR "children with medical complex*" OR "children with special health care need?" OR "children with special health care need?" OR "chronic health conditions in childhood" OR "co-morb*" OR "complex care" OR "complex chronic condition?" OR "complex health need?" OR "development* disab*" OR disab* OR "life-limiting condition?" OR "life-long care" OR "life-long condition?" OR "life-long disease?" OR "life-long illness" OR "life-long impairment" OR "life-threatening condition?" OR "life-threatening disease?" OR "long term care" OR "long term condition?" OR "long term disease?" OR "long term health" OR "long

		term illness" OR "long term impairment" OR "long term psych*" OR "long term mental issue?" OR "long term mental condition?" OR "long term mental disease?" OR "long term psych*" OR "medical technology dependent" OR "medical complex*" OR "multi-morb*" OR "profound intellectual and multiple disabilities" OR "Severe motor and intellectual disabilities" OR "severe motor and intellectual disabilities medical care dependent group" OR "special health* need*" OR "special health care need?" OR "special need*" OR "Special Needs Kids" OR "SpeNK" OR "CSHCN" OR "CMC" OR "CCMC" OR "CYSHCN" OR "PIMD" OR "SMID" OR "SMID-MCDG"
	**C. Integrated Care Model**	"care continuity" OR "care coordination" OR "care framework" OR "care pathway" OR "care planning" OR "care transition" OR "case management" OR "care model" OR "child health services" OR "chronic care model" OR "collaborative care" OR "continuity of care" OR "cooperative care" OR "coordinated care" OR "coordinated care model" OR "disease management" OR "horizontal integration" OR "integrate* care" OR "interdisciplinary" OR "interprofessional" OR "intersectoral care" OR "intrasectoral care" OR "linked care" OR "longitudinal integration" OR "managed care" OR "medical home" OR "multi sector" OR "multiagency" OR "multidisciplinary" OR "multiprofessional" OR "patient centered" OR "self-care" OR "service network" OR "shared care" OR "transitional care" OR "vertical integration" OR "virtual integration" OR "whole system thinking"

	**D. Randomized controlled trials**	1 exp randomized controlled trial/<br>2 Controlled clinical trial/<br>3 random$.ti,ab.<br>4 randomization/<br>5 intermethod comparison/<br>6 placebo.ti,ab.<br>7 (compare or compared or comparison).ti.<br>8 ((evaluated or evaluate or evaluating or assessed or assess) and (compare or compared or comparing or comparison)).ab.<br>9 (open adj label).ti,ab.<br>10 ((double or single or doubly or singly) adj (blind or blinded or blindly)).ti,ab.<br>11 double blind procedure/<br>12 parallel group$1.ti,ab.<br>13 (crossover or cross over).ti,ab.<br>14 ((assign$ or match or matched or allocation) adj5 (alternate or group$1 or intervention$1 or patient$1 or subject$1 or participant$1)).ti,ab.<br>15 (assigned or allocated).ti,ab.<br>16 (controlled adj7 (study or design or trial)).ti,ab.<br>17 (volunteer or volunteers).ti,ab.<br>18 human experiment/<br>19 trial.ti.<br>20 or/1-19

		21 (random$ adj sampl$ adj7 ("cross section$" or questionnaire$1 or survey$ or database$1)).ti,ab. not (comparative study/ or controlled study/ or randomi?ed controlled.ti,ab. or randomly assigned.ti,ab.)<br>22 Cross-sectional study/ not (exp randomized controlled trial/ or controlled clinical study/ or controlled study/ or randomi?ed controlled.ti,ab. or control group$1.ti,ab.)<br>23 (((case adj control$) and random$) not randomi?ed controlled).ti,ab.<br>24 Systematic review.ti,ab. not (trial or study).ti.<br>25 (nonrandom$ not random$).ti,ab.<br>26 "random field$".ti,ab.<br>27 (random cluster adj3 sampl$).ti,ab.<br>28 (review.ab. and review.pt.) not trial.ti.<br>29 "we searched".ab. and (review.ti. or review.pt.)<br>30 "update review".ab.<br>31 (databases adj4 searched).ab.<br>32 (rat or rats or mouse or mice or swine or porcine or murine or sheep or lambs or pigs or piglets or rabbit or rabbits or cat or cats or dog or dogs or cattle or bovine or monkey or monkeys or trout or marmoset$1).ti. and animal experiment/<br>33 Animal experiment/ not (human experiment/ or human/)<br>34 or/21-33<br>35 20 not 34

Database: Ovid Medline(R) <1946 to 2023 May 30>

AND	**A. Target group**	adolescen* OR "Adolescents and Young Adults" OR "AYA" OR bab* OR child* OR infan* OR minor? OR neonat* OR p?ediatric OR puberty OR pubescent OR teen OR young* OR youth OR "young people"
	**B. Complex/ special care needs/ Multi/ comorbidity/disability**	"chronic care" OR "chronic condition?" OR "chronic disab*" OR "chronic disease?" OR "chronic health issue?" OR "chronic illness" OR "chronic impairment" OR "chronic mental issue?" OR "chronic mental condition?" OR "chronic mental disease?" OR "chronic psych*" OR "child disab*" OR "child medically fragile" OR "children with medical complex*" OR "children with special health care need?" OR "children with special health care need?" OR "chronic health conditions in childhood" OR "co-morb*" OR "complex care" OR "complex chronic condition?" OR "complex health need?" OR "development* disab*" OR disab* OR "life-limiting condition?" OR "life-long care" OR "life-long condition?" OR "life-long disease?" OR "life-long illness" OR "life-long impairment" OR "life-threatening condition?" OR "life-threatening disease?" OR "long term care" OR "long term condition?" OR "long term disease?" OR "long term health" OR "long term illness" OR "long term impairment" OR "long term psych*" OR "long term mental issue?" OR "long term mental condition?" OR "long term mental disease?" OR "long term psych*" OR "medical technology dependent" OR "medical complex*" OR "multi-morb*" OR "profound intellectual and multiple disabilities" OR "Severe motor and intellectual disabilities" OR "severe motor and intellectual disabilities medical care dependent group" OR "special health* need*" OR "special health care

		need?" OR "special need*" OR "Special Needs Kids" OR "SpeNK" OR "CSHCN" OR "CMC" OR "CCMC" OR "CYSHCN" OR "PIMD" OR "SMID" OR "SMID-MCDG"
	**C. Integrated Care Model**	"care continuity" OR "care coordination" OR "care framework" OR "care pathway" OR "care planning" OR "care transition" OR "case management" OR "care model" OR "child health services" OR "chronic care model" OR "collaborative care" OR "continuity of care" OR "cooperative care" OR "coordinated care" OR "coordinated care model" OR "disease management" OR "horizontal integration" OR "integrate* care" OR "interdisciplinary" OR "interprofessional" OR "intersectoral care" OR "intrasectoral care" OR "linked care" OR "longitudinal integration" OR "managed care" OR "medical home" OR "multi sector" OR "multiagency" OR "multidisciplinary" OR "multiprofessional" OR "patient centered" OR "self-care" OR "service network" OR "shared care" OR "transitional care" OR "vertical integration" OR "virtual integration" OR "whole system thinking"
	**D. Randomized controlled trials**	1 Randomized controlled trials as Topic/<br>2 Randomized controlled trial/<br>3 Random allocation/<br>4 Double blind method/<br>5 Single blind method/<br>6 Clinical trial/<br>7 exp Clinical Trials as Topic/<br>8 or/1-7<br>9 (clinic$ adj trial$1).tw.

		10 ((singl$ or doubl$ or treb$ or tripl$) adj (blind$3 or mask$3)).tw.<br>11 Placebos/<br>12 Placebo$.tw.<br>13 Randomly allocated.tw.<br>14 (allocated adj2 random).tw.<br>15 or/9-14<br>16 8 or 15<br>17 Case report.tw.<br>18 Letter/<br>19 Historical article/<br>20 Review of reported cases.pt.<br>21 Review, multicase.pt.<br>22 or/17-21<br>23 16 not 22
