# Supplementary material for: Integrated care for children and young people with special health and care needs: a systematic review
Source: Arch Dis Child. 2024 Jul 30;109(11):e326905. doi: 10.1136/archdischild-2024-326905 (PMC11503122; doi:10.1136/archdischild-2024-326905)
Supplement: online supplemental table 1 [file archdischild-109-11-s002.docx]

**Table 1a. Details of characteristics of studies, outcomes, and interventions**

| **Study  (Author  year)** | Beyene et.al. 2013^20^ | Braga *et.al.* 2005^33^ | Bruns *et.al.* 2015^26^ | Cady  *et.al.* 2015^21^ | Carcone *et.al.* 2015^30^ | Caskey *et.al.* 2019^27^ | Cohen  *et al.* 2023^25^ | Coller  *et.al.* 2018^22^ | Farmer *et.al.* 2011^23^ | Frakking  *et al.* 2021^28^ | Gillette *et.al.* 1991^31^ | Jessop  *et.al.* 1994^24^ | Kolko  *et.al.* 2020^32^ | Simon  *et.al.* 2017^29^ |
| --- | --- | --- | --- | --- | --- | --- | --- | --- | --- | --- | --- | --- | --- | --- |
| **Setting** | USA | Brazil | USA | USA | USA | USA | Canada | USA | USA | Australia | USA | USA | USA | USA |
| **Participants (N): Age range (years)  INT  CON** | 445  0-19y  148M, 81F 149M, 67F | 72 5-12y  20M, 18F 19M, 15F | 93 6-17y  26M, 21F 27M, 19F | 163 2-15y  INT 1: 33M, 21F INT 2: 26M, 28F CON: 31M, 24F | 146 10-17y  32M, 42F 32M, 40F | 6259 0-25y  1670M, 1456F 1456M, 1663F | 139 0-9y  45M, 32F 38M, 24F | 147 0-8y  43M, 34F 44M, 26F | 70 0-17y  23M, 13F 23M, 11F | 81 0-16y  28M, 14F 27M, 12F | 38 0-1y  15M, 4F 15M, 4F | 174 0-11y  NR NR | 206 5-12y  72M, 28F 70M, 36F | 331 0-18y  89M, 70F  79M, 55F |
| **Chronic and complex condition(s) included in study** | **Multiple disorders:** Cerebral palsy, Brain injury, developmental difficulties, Down syndrome, Spina bifida, Autism, Physical disability, Developmental disability, Pervasive developmental disorder, or a chronic medical condition | Traumatic brain injury (TBI) | **Multiple disorders:** mental, behavioural or emotional disorder | **Multiple disorders:** Single complex chronic condition, Multiple complex chronic conditions, Neurologic impairment, Need for life-sustaining technology assistance | Type 1 diabetes (T1D) and Type 2 diabetes (T2D) | **Multiple disorders:** Asthma, diabetes (type 1 or 2), prematurity, seizure disorder, or sickle cell disease | **Multiple disorders** Children with Medical complexity (CMC) | **Multiple disorders** Children with Medical complexity (CMC) | **Multiple disorders:** Mental disorders, Disease of nervous system/sense organs; Congenital anomalies, Perinatal conditions, Organ specific conditions | **Multiple disorders:** Attention-deficit/hyperactivity  Disorder (ADHD), autism spectrum disorder, global developmental delay, oppositional defiant  disorder, adjustment disorder, and generalized anxiety disorder | Medically Fragile Infants | **Multiple disorders:** Children had >100 different conditions, including sickle cell anaemia, asthma, diabetes, leukemia, juvenile rheumatoid arthritis, and congenital conditions such as meningomyelocele and biliary atresia | Behavior problems with comorbid ADHD | **Multiple disorders** Children with Medical complexity (CMC) |
| **Risk of bias (ROB) score** | Moderate | High | High | Low | High | Low | Low | Moderate | Moderate | Low | High | Moderate | Moderate | Moderate |
| **OUTCOMES** |  |  |  |  |  |  |  |  |  |  |  |  |  |  |
| 1. **Child health and functioning** |  |  |  |  |  |  |  |  |  |  |  |  |  |  |
| - 1. **Physical (motor) functioning**  *GRADE score* |  | (p=0.018) ^a^  *Low* |  |  |  |  |  |  |  |  | (p=0.5) ^b^  *Low* |  |  |  |
| - 1. **Cognitive functioning**  *GRADE score* |  | (p=0.05) ^c^  *Very low* |  |  |  |  |  |  |  |  | (p=0.24) ^d^  *Very low* |  |  |  |
| - 1. **Behavioural functioning**  *GRADE score* |  |  | (p=.078) ^e^  *Very low* |  |  |  |  |  |  |  |  |  |  |  |
| - 1. **Youth functioning**  *GRADE score* |  |  | (p=0.710) ^f^ *Very low* |  |  |  |  |  |  |  |  |  |  |  |
| - 1. **Child’s functional status**  *GRADE score* |  |  |  |  |  |  |  |  | (p=0.06) ^g1^  *Very low* |  |  |  |  | (T1 & T2; p>0.05) ^g2^  *Very low* |
| - 1. **Physical pain**  *GRADE score* |  |  |  |  |  |  | (T1 - p = 0.86) ^h^ (T2 - p = 0.87)  *Very low* |  |  |  |  |  |  |  |
| 1. **Parent/carer perception on child health & family wellbeing** |  |  |  |  |  |  |  |  |  |  |  |  |  |  |
| - 1. **Quality of Life**  *GRADE score* | (p = 0.32) ^i^     Very low |  |  |  |  |  | (T1-p=0.03) ^¶^  (T2-p=0.28) *Very low* |  |  | (T1-p=0.06)^i^   (T2-p=0.04)  *Very low* |  |  | (T1-p=0.03)^i^ (T2-p=0.02)   *Very low* |  |
| - 1. **Family functioning**  *GRADE score* | (p=0.97) ^j^ *Very low* |  |  |  |  |  |  |  | (p=0.69) ^k^ *Very low* | (T1-p=0.09)^l^ (T2-p=0.004) *Very low* |  |  |  |  |
| - 1. **Parental distress** *GRADE score* | (p=0.09) ^m^  *Very low* |  |  |  |  |  | (T1-p =0.03) ^n^ (T2-p=0.14)  *Very low* |  | (p=0.07) ^o^  *Very low* | (T1 & T2; p>0.05) ^p^ *Very low* |  |  |  |  |
| - 1. **Social support**  *GRADE score* | (p=0.86) ^q^ *Very low* |  |  |  |  |  |  |  | (p=0.75) ^r^ *Very low* |  |  |  |  |  |
| - 1. **Satisfaction with life**  *GRADE score* |  |  |  |  |  |  | (T1-p=0.04) **^s^** (T2-p=0.29) *Very low* |  |  |  |  |  |  |  |
| - 1. **Fatigue**  *GRADE score* |  |  |  |  |  |  | (T1-p =0.05) **^t^** (T2-p =0.09) *Very low* |  |  |  |  |  |  |  |
| - 1. **Sleep disturbance**  *GRADE score* |  |  |  |  |  |  | (T1-p=0.08) ^u^ (T2-p=0.003) *Very low* |  |  |  |  |  |  |  |
| - 1. **Physical health** *GRADE score* |  |  |  |  |  |  | (T1-p=0.16) ^v^ (T2-p=0.01) Very low |  |  |  |  |  |  |  |
| 1. **Healthcare use &spend**  *Hospitalization     Total charges      GRADE score* |  |  |  |  |  | (p=0.85) ^w^    (p=0.99) ^x^  *Very low* | (T1- p=0.82) ^w^ (T2- p=0.73)  (T1- p=0.90) **^x^** (T2- p=0.01)  *Very low* | (p=0.04) ^w^   (p=0.02) ^x^  *Very low* |  |  |  |  |  | (T1 & T2; p>0.05) ^w^ (T1-p=0.09) ^x^ (T2-p=0.01) ^x^  *Very low* |
| 1. **Care coordination**  *GRADE score* | NR | NR | NR | (p<0.05) ^y^ *Very low* | (p <0.05)^z^ *Very low* |  | HCP^$^ (T1-p=0.44), (T2-p=0.55)  HCP&F^¥^ (T1-p=0.08) (T2-p=0.46)  *Very low* | NR | (T1-p=0.058) ^€^  (T2-p=0.0001)  *Very low* |  | (p<0.001)^$^  *Very low* | (p<0.05) ^£^  *Very low* |  | (T1-p=0.05) (T2-p=0.004) ^✞^  *Very low* |
| **INTERVENTION** |  | | | | | | | | | | | | |  |
| **Description of integrated care/care-coordination** | Children's Treatment Network: an integrative health team together with family members develop a tailored single plan of care for the child, through ***a service coordinator.*** | Indirect, family-supported treatment (IFS): to provide families the skills to deliver home based care through 2 case managers with appropriate specializations to train families and acting ***as service navigators***. | Wraparound service of individualized, team-based holistic care planning through wraparound facilitators acting as ***service navigators***. | A single clinic-based ***Advanced Practice Registered Nurse (APRN) care coordinator*** | Multisystemic Therapy (MST):an intensive, home and community-based family treatment (psychotherapy approach), delivered by a ***psychotherapist*** functioning as a care coordinator | CHECK (Coordinated HEalthcare for Complex Kids): quality improvement project of comprehensive community-based care for children and young adults with care coordination delivered by ***community health workers***. | Complex Care for Kids Ontario (CCKO): Assignment of a **nurse practitioner–paediatrician dyad** partnering with families in a structured complex care clinic to provide intensive care coordination and comprehensive plans of care. | Plans for Action and Care Transitions (PACT), delivered by multidisciplinary team, created by ***a medical home physician or nurse practitioner.*** | ***Family Support Specialist*** (FSS): liaising with primary care, specialty health care providers and community service agencies to improve access to and coordination of comprehensive care. | Care coordination delivered by an **Allied Health Liaison Officer (AHLO).** | CATCH: A Collaborative Approach to the Transition from the Hospital to the Community and Home through a collaboration of ***family and the community professional***. | Paediatric Home Care (PHC): a comprehensive outreach program in which a ***multidisciplinary team*** of paediatricians, paediatric nurse practitioners, and a social worker deliver comprehensive services, including case management. | Doctor Office Collaborative Care (DOCC) - Children and parents received on-site services in coordination with the Primary Care Providers (PCP) through ***care managers.*** | Seattle Children’s Hospital (SCH) developed a comprehensive case management service (CCMS), a multidisciplinary, hospital-based service focused on improving care coordination for CMC. |
| WHO integrated care model category | Managed clinical network | Case-management | Care planning | Care planning | Care planning | Disease-specific integrated care model | Managed clinical network | Care planning | Care planning | Care planning | Case-management | Patient-centered medical home | Case-management | Case-management |

**Note:**

= no difference; = improvement in outcome; = decline in outcome.

^a^ = SARAH Scale of Motor Development: 12 months follow-up
^b^= Bayley Psychomotor Index (PDI): 6 months follow-up
^c^ = Weschsler Intelligence Scale for Children (WISC-III): 12 months follow-up
^d^ = Bayley Mental Developmental Index (MDI): 6 months follow-up

^e^ = Strengths and Difficulties Questionnaire (SDQ): 6-12 months follow-up
^f^ = The Child and Adolescent Functional Assessment Scale (CAFAS): 6-12 months follow-up
^g1&2^ = Child functional status (FS II-R)^:^ follow-up: 6 months ^g1^ and 12-18 months ^g2
h^ = A 10-cm linear visual analog scale at 12 months (T1) and 24 months (T2) after intervention
^i^ = Pediatric Quality of Life Inventory (PedsQL): follow-up time points: 6, 12, 18 and 24 months.

^¶^ = KIDSCREEN-52: follow-up at 12 months (T1) and 24 months (T2)
^j^ = Byles J, Byrne C, Boyle MH, Offord DR. Ontario Child Health Study: reliability and validity of the general functioning subscale of the McMas­ter Family Assessment Device. *Fam Process*. 1988;27(1):97–104.
^k^ = Bailey, D. B., & Simeonsson, R. J. (1988). Assessing needs of families with handicapped infants. Journal of Special Education, 22, 117–126.
^l^ = Family functioning subscale from the PedsQL Family Impact Module, version 2.0 at 12 months (T1) and 24 months (T2) after intervention
^m^ = Kessler RC, McGonagle KA, Zhao S, et al. Lifetime and 12-month prevalence of DSM-III-R psychiatric disorders in the United States: results from the National Comorbidity Survey. *Arch Gen Psychiatry*. 1994;51(1):8–19.
^n^ = Patient Reported Outcomes Measurement Information System at 12 months (T1) and 24 months (T2) after intervention
^o^ = Beck Depression Inventory-II (BDI-II) : follow-up: range 6 to 24 months
^p^ = Subjective Units of Distress Scale (SUDS) at 6 months (T1) and 12 months (T2) after intervention
^q^ = Cutrona CE, Russell DW. The provisions of social relationships and adaptation to stress. *Advances in Personal Relationships*. 1987;1(1): 37–67.
^r^ = Dunst, C. J., Trivette, C. M., & Hamby, D. W. (1994). Measuring social support in families with young children with disabilities. In C. J. Dunst, C. M. Trivette, & A. J. Deal (Eds.), Supporting and strengthening families: Methods, strategies, and practices. Cambridge, MA: Brookline Books
^s^ = Satisfaction With Life Scale at 12 months (T1) and 24 months (T2) after intervention
^t^ = Patient Reported Outcomes Measurement Information System at 12 months (T1) and 24 months (T2) after intervention
^u^ = Patient Reported Outcomes Measurement Information System at 12 months (T1) and 24 months (T2) after intervention
^w^ = Rate of hospitalization
^x^ = Total patient charges
^y^ = Survey question US Department of Health and Human Services (USDHHS). (2008). Health Resources and Services Administration, Maternal and Child Health Bureau. National Survey of Children with Special Health Care Needs 2005–2006
^z^ = King S, King G, Rosenbaum P. Evaluating health service delivery to children with chronic conditions and their families: Development of a refined measure of processes of care (MPOC-20). Children's Health Care. 2004; 33:35–57.
^$^ = Coordination of care among health care professionals (HCP) using Family Experience With Care Coordination (FECC) scores (FECC 8a & 8b) at 12 months (T1) and 24 months (T2) after intervention
^¥^ = Coordination of care among HCP & Family (F) using Family Experience With Care Coordination (FECC) scores (FECC 5) at 12 months (T1) and 24 months (T2) after intervention
^€^ = Epstein, S. G., Taylor, A. B., Halberg, A. S., Gardner, J. D., Walker, D. K., & Crocker, A. C. (1998). Shared responsibilities: Ensuring quality managed care for children with special health care needs. Took kit: Version 1.0. Boston, MA: New England.
^$ & £^= Structured interview questions
^✞=^ Consumer Assessment of Healthcare Providers and Systems (CAHPS) version 4.0 Child Health Plan Survey
